# Venetoclax: A New Partner in the Novel Treatment Era for Acute Myeloid Leukemia and Myelodysplastic Syndrome

**DOI:** 10.1007/s44228-023-00041-x

**Published:** 2023-04-18

**Authors:** Jean El-Cheikh, Ghassan Bidaoui, Mustafa Saleh, Nour Moukalled, Iman Abou Dalle, Ali Bazarbachi

**Affiliations:** 1 Division of Hematology/Oncology, Department of Internal Medicine, American University of Beirut Medical Center, Beirut, Lebanon; 2 Faculty of Medical Sciences, Lebanese University, Beirut, Lebanon; 3 Department of Internal Medicine, Medical Center, Bone Marrow Transplantation Program, American University of Beirut, P.O. Box 113-6044, Beirut, Lebanon

**Keywords:** Venetoclax, Acute myeloid leukemia, Myelodysplastic syndrome, Targeted therapy, Apoptosis, Bcl-2, Hypomethylating agents, Myeloid neoplasia

## Abstract

**Background:**

Acute Myeloid Leukemia (AML) and Myelodysplastic Syndrome (MDS) are two closely related blood cancers that are more frequent in older adults. AML is the most common type of adult acute leukemia, and MDS is characterized by ineffective blood cell production and abnormalities in the bone marrow and blood. Both can be resistant to treatment, often due to dysfunction in the process of apoptosis, the body’s natural mechanism for cell death. Venetoclax, an orally-administered medication that selectively targets the BCL-2 protein, has shown promise in enhancing treatment sensitivity in some hematological malignancies by reducing the apoptotic threshold. This review aims to evaluate the effectiveness of venetoclax in treating AML and MDS, as well as potential mechanisms of resistance to the medication.

**Methods:**

A literature search was conducted utilizing PUBMED to capture all relevant research articles on the use of venetoclax as a therapy for both diseases. The MeSH terms “acute myeloid leukemia”, “myelodysplastic syndrome” and “venetoclax” were searched. Furthermore, Clinicaltrials.gov was accessed to ensure the inclusion of all ongoing clinical trials.

**Results:**

Although Venetoclax showed modest results as a single-agent therapy in AML, venetoclax-based combination therapies? mainly with hypomethylating agents or low-dose cytarabine? yielded significantly positive results. Preliminary results oN the use of venetoclax-based combination therapy with HMA, mainly azacitidine, in unfit high-risk MDS also yielded optimistic results. Identification of mutations for which various drugs have been approved has spurred active investigation of venetoclax in combination trials.

**Conclusion:**

Venetoclax-based combination therapies have been shown to induce rapid responses and increase overall survival in AML patients unfit for intensive chemotherapy. These therapies are also yielding positive preliminary results in high-risk MDS patients in phase I trials. Resistance to venetoclax and drug-related toxicity are two main obstacles that need to be overcome to reap the full benefits of this therapy.

## Introduction

### Acute Myeloid Leukemia

Acute myeloid leukemia (AML) is the most common form of acute leukemia, with the median age of presentation being 68 years [1, 2]. The management of AML poses a complex challenge, due to the disease’s heterogeneous nature and the advanced age of most patients. To determine the optimal strategy for patients with AML, a variety of factors must be taken into consideration, including the patient's suitability for intensive chemotherapy (IC) and allogeneic stem cell transplantation (allo-SCT), age, and the unique characteristics of their disease such as karyotype and somatic mutations [3]. Risk stratification models, such as the ALFA classifier, ELN 2017, and ELN 2022 (ELN-22), are used to guide treatment decisions. Among these, the ELN-22 classifier has emerged as the most effective tool for prognostic scoring in AML patients. The ELN-22 classifier categorizes patients into favorable-, intermediate-, and adverse-risk groups [4].

The standard therapy for patients under 65 years of age who are fit for IC is typically a combination of the antimetabolite cytarabine and an anthracycline in the 7 + 3 regimen. This combination has been shown to yield a complete remission (CR) rate of 60 to 80% with a median overall survival (OS) of 16 to 24 months in younger patients [5, 6]. Despite the potential for dose augmentation and the addition of other chemotherapy agents to improve CR rates to 80%, unfortunately, 40% of patients still relapse [1]. FLT3-mutated AML, classified as an intermediate-risk entity according to the ELN-22 classification system, has traditionally been associated with poor prognosis. However, the introduction of FLT3 tyrosine kinase inhibitors has significantly improved outcomes for patients with this subtype of AML. This shift in prognosis, secondary to the introduction to a targeted therapy, highlights the potential of the latter, including venetoclax, in leukemia patients [7–9].

In the realm of AML and myelodysplastic syndromes (MDS), hypomethylating agents (HMA) or low-dose cytarabine (LDAC) are considered the standard of care for patients older than 65 years or those unfit for IC [10, 11]. Patients receiving azacitidine showed a median OS of 12.1 months and an overall response rate (ORR) of 27.8%, compared to an OS of 6.5 months and an ORR of 25.1% for those receiving conventional regimens. This represents a 4.8-months increase in median OS compared to LDAC [12]. Furthermore, for patients with relapsed or refractory (R/R AML), the prognosis is typically poor, with ORR ranging from 4 to 16% and median OS of 2–7 months [13, 14]. It is important to note that given the median age of presentation of both AML and MDS, the majority of the patients are unfit for IC and allo-SCT. Consequent to the aforementioned outcomes and limitations, preclinical and clinical trials throughout the last three decades focused on elucidating the molecular and genetic basis of the development of resistance to therapy inherent to AML, with the hopes of increasing the arsenal of treatment for unfit patients [1].

Allo-SCT continues to gain momentum as a powerful tool in preventing relapse for AML patients. Thanks to algorithms, physicians are now able to identify those patients who would most greatly benefit from allo-SCT in their first CR through genetic and measurable residual disease analysis [15]. Studies consistently demonstrate that patients who receive allo-SCT while still in active disease have poorer outcomes than those who are treated in CR [16]. However, patients who achieve a complete remission with incomplete count recovery (CRi) or partial remission (PR) after treatment still have a chance to achieve a cure through allo-SCT. Furthermore, despite a higher risk of non-relapse mortality, such patients do not necessarily exhibit an elevated risk of relapse [17, 18]. The goal of therapy for fit adults with R/R AML is to proceed to allo-SCT once a second CR has been achieved and, fortunately, there are now prognostic systems to identify patients with good long-term survival prospects [19, 20]. Retrospective analyses of allo-SCT for AML in CR2 have demonstrated OS rates of 30–60% with acceptable rates of treatment-related mortality [21].

Targeted therapies have already had a significant impact in the treatment of AML, improving survival and quality of life for patients. However, the genomic complexity of AML poses a significant challenge in achieving accurate risk stratification and targeted therapy [22]. Despite this, new agents such as SMO inhibitors, immune checkpoint inhibitors, metabolic and pro-apoptotic agents, and monoclonal or bispecific T-cell engager antibodies are being developed and investigated as potential therapies for AML [23]. It is worth to mention that the discovery of IDH inhibitors such as Ivosidenib and Enasidenib, specifically targeting the IDH1 and IDH2 proteins, respectively, has opened up new opportunities for targeted therapy in AML, showing great promise for improving patient outcomes [22, 24]. Furthermore, Ivosidenib has demonstrated substantial efficacy in a small subgroup of IDH1-mutated MDS patients refractory to therapy with HMA [25].

### Myelodysplastic Syndrome

MDS is also a heterogeneous hematological malignancy, which shares many similarities to AML’s pathobiology and outcomes. Patients with MDS are often older at diagnosis and present with a variety of symptoms, such as low levels of one or more blood cells, and an abnormal number of cells in the bone marrow [26, 27]. The underlying causes of MDS are complex and involve an interplay of alterations in the chromosomes, genetic mutations, and a pro-inflammatory microenvironment. Despite the heterogeneity of MDS, researchers have developed risk assessment scores such as the Revised International Prognostic Scoring System to help guide the treatment [26–29]. Low-risk MDS can be treated with only observation if the patient is transfusion-independent, but also with lenalidomide (5q mutation), transfusions, iron chelation, and/or erythropoietin, as needed [11, 14]. High-risk MDS (HR-MDS) is usually treated with HMA or AML-like chemotherapy, as a bridge to the only curative option, allo-SCT. Unfit HR-MDS patients, who comprise a large portion of HR-MDS patients, should receive HMA, preferably azacytidine, given its proven OS benefit in these patients, and should be advised to join clinical trials [11, 12, 14].

### Intrinsic Apoptosis Pathway and Venetoclax

The intrinsic apoptosis pathway is a highly conserved pathway that is highly regulated by the balance between pro-apoptotic and anti-apoptotic molecules [30, 31]. The former include BAX, BAK, BIM, BID, and PUMA, while the main anti-apoptotic molecules are BCL-2, BCL Xl, MCL-1, and MDM2. BAX and BAK are the effectors of apoptosis and are activated by the other BH3 pro-apoptotic molecules such as BID. The BH3 molecules are upregulated as a consequence to cell stress, which is signaled by activated TP53. When activated, BAX and BAK increase mitochondrial outer membrane permeability, which leads to a cytochrome release [30, 31]. Cytochrome C induces the activation of caspase 9. Anti-apoptotic molecules usually inhibit apoptosis by sequestering the BH3 molecules, inhibiting the activation of BAX and BAK [30, 31].

Venetoclax is an effective BH3 mimicker and a highly selective oral BCL-2 inhibitor that showed transformative efficacy in the treatment of chronic lymphocytic leukemia (CLL) [32, 33]. Based on the results of the MURANO and the CLL14 trials, venetoclax plus anti-CD20 antibody became the standard of care for naïve and R/R CLL in many instances [34–37]. Venetoclax binds the anti-apoptotic protein BCL-2 at the BH3-only pro-apoptotic pocket, displacing BH3 pro-apoptotic molecules. These pro-apoptotic molecules are then free to activate intrinsic apoptosis effectors. Although not as homogeneously as in CLL, BCL-2 is highly expressed in leukemic stem cells in AML and MDS. Hence, studies on the efficacy of Venetoclax in AML patients and then MDS patients emerged [34].

### Venetoclax Studies on AML Patients

Venetoclax monotherapy in AML patients yielded unsatisfactory results (Table 1). The first single-agent phase II trial of venetoclax on 32 R/R AML patients yielded an ORR of 19%, CR of 6%, and a median OS of 4.7 months. It gave insight, however, into the possibility of BCL-2 dependence and apoptosis dysfunction, as is the case with CLL [34, 38]. Hence, trials on combination therapy have been done [27]. Venetoclax-based combination therapy showed significant results in AML patients [34, 38]. One main trial is the phase III VIALE-A. Newly diagnosed (ND) unfit AML patients were randomly distributed to receive either venetoclax plus azacytidine or placebo plus azacytidine. The median OS turned out to be 14.7 months versus 9.6 months in favor of the venetoclax arm. The difference in ORR was also significant, with 66.4% versus 28.3% in favor of the venetoclax arm [32–34, 38, 39]. Venetoclax-based combination regimens with azacytidine, decitabine, FLAG-IDA or LDAC led to deep response, and even clinically significant OS prolongation, in ND AML cases in multiple trials [39–45]. Based on those results, The US Food and Drug Administration approved venetoclax with HMA agents for patients unfit for IC and those older than 75 years in 2018, with full approval in October 2020 [34, 39].

**Table 1:** List of published clinical trials assessing venetoclax in association with other agents in AML and MDS

Authors	Agents	Population	Median PFS (in months)	Median OS (in months) (Median Follow up in months)	ORR	CR	Patients enrolled	Phase study
Konopleva et al. [38]	Venotoclax	AML unfit for IC	2.3	4.7 (117 d)	19%	6%	32	II
Garcia JS et al. [65]	Venetoclax + AZA	Rx-naïve HR-MDS	–	27.5	77%	42%	78	Ib
Di Nardo et al. [39]	Venetoclax + AZA (VIALE-A trial)	AML unfit for IC	9.8	14.7 (20.5)	66.70%	36.70%	576	III
Pollyea et al. [40]	Venetoclax + AZA or DEC	AML unfit for IC	–	16.4 (29) and 16.2 (40)	–	44% and 55%	115	Ib
Yamamoto et al. [45]	Venetoclax + AZA (VIALE-A trial Japan subrgoup)	AML unfit for IC	16.3	NR (16.3)	–	46%	37	III
Ball et al. [66]	Venetoclax + HMAs	Rx-naive and R/R MDS	15.4	19.5 (7.6)	59%	14%	44	Ib
Wei et al. [42]	Venetoclax + LDAC (VIALE-C trial)	AML unfit for IC	4.9	8.4 (17.5)	–	28%	211	III
Hu et al. [43]	Venetoclax + LDAC	AML unfit for IC	–	9	–	33%	15	III
*Intensive chemotherapy*								
Vives et al. [46]	Ventoclax + AZA or FLUGA	ND AML	4.9 & 3.0	9.8 (29.1) and 4.1 (32.9)	23% 33%	9% and 18%	283	III
Kadia et al. [47]	Venotoclax + CLIA regimen	ND AML and HR-MDS	NR	NR (13.5)	95%	85%	50	II
Chua et al. [48]	Venotoclax + (5 + 2)	ND AML ≥ 64 yo	–	11.2 (22.9)	72%	41%	51	Ib
Di Nardo et al. [41]	Venotoclax + FLAG-IDA	ND and R/R-AML	NR	NR (12)	–	69% and 48%	68	Ib/II
*Non-Intensive chemotherapy*								
Daver et al. [76]	Venetoclax + GILT	R/R FLT3 mutated AML	–	10 (17.5)	–	81%	61	Ib
Short et al. [77]	Venetoclax + AZA + GILT	R/R and ND AML	–	10.5 (9.5) and NR (3.8)	67% and –	7% and 73%	26	I/II
Yilmaz et al. [78]	Venetoclax + LIC + FLT3i	ND or R/R FLT3 mutated AML	–	NR (12)	–	67%	87	II
Lachowiez et al. [79]	Venetoclax + IVO + AZA	MDS, ND or R/R AML IDH1 +	NR	NR (16.1)	92%	–	25	Ib/II
Chan et al. [80]	Venetoclax + ENA	MDS or R/R AML IDH2 +	–	NR (3.5)	55%	22%	11	Ib/II
Daver et al. [81]	Venetoclax + AZA + MAGRO	R/R AML and ND AML IC ineligible	–	–	–	81%	38	I/II
Borthakur et al. [82]	Venetoclax + MIVE (pan-BETi)	R/R AML	11.8 weeks	37.4 weeks	–	7%	44	I
Daver et al. [83]	Venetoclax + IDASA	R/R AML ≥ 60 yo	–	4.4 (3.4)	41%	–	49	Ib
Daver et al. [84]	Venetoclax + COB (MEKi)	R/R AML ≥ 60 yo, IC ineligible	–	–	18%		42	Ib

*AZA* azacytidine, *BETi* BET inhibitor, *DEC* decitabine, *LDAC* low dose cytarabine, *FLUGA* semi-intensive regimen with LDAC plus oral fludarabine, *CLIA* cladribine, high-dose cytarabine, idarubicin, *FLAG* fludarabine, *IDA* idarubicin, *GILT* gilteritinib, *LIC* Low intensive chemotherapy, *FLT3i* FLT3 inhibitors, *IVO* ivosidenib, *ENA* enasidenib, *MAGRO* magrolimab, *MIVE* mivebresib, *IDASA* idasanutlin, *COB* cobimetinib, *MEKi* MEK inhibitor, *pan-BETi* non-selective BET inhibitors, *IC* intensice chemotherapy, *ND AML* newly diagnosed acute myeloid leukemia, *HR-MDS* high risk myelodysplastic syndrome

Venetoclax is being evaluated as a treatment for younger patients with AML and various IC regimens [46]. While no published data exist on the use of the standard IC regimen (7 + 3) with venetoclax, several clinical trials are underway or about to begin (NCT03709758 and NCT04628026). One phase II trial at the MD Anderson Cancer Center combined venetoclax with CLIA (cladribine, high-dose cytarabine, and idarubicin) in AML patients and achieved a composite CR rate of 94%, with 82% having undetectable minimal residual disease (MRD) [47]. In a phase Ib study, venetoclax was paired with a 5 + 2 regimen in elderly AML patients who were ineligible for IC, resulting in a 72% CR/CRi rate and a median OS of 11 months [48]. Another phase Ib/II trial of venetoclax in combination with FLAG-IDA in ND and R/R AML showed a CR rate of 88%, with 56% of patients bridged to allo-SCT [41].

These significant results in unfit AML patients led to retrospective studies that explored the possibility of using venetoclax-based regimens before and after allo-SCT as salvage therapy. One such study was done at Memorial Sloan Kettering Cancer Center and Yale University [49]. For the 39 patients who received venetoclax-based combination therapy before allo-SCT, the median OS was not reached. However, the 12-months OS was 79%. The 12-months OS of patients who received venetoclax post-transplant as salvage therapy was 43.4%, with 4 patients undergoing a second allo-SCT [49]. These results highlight the possible benefit of adding venetoclax to IC before allo-SCT to induce remission. They also highlight a possible advantage of using these regimens after allo-SCT in the case of relapse, as salvage therapy. For example, in cases of R/R AML patients, it is still possible to proceed with allo-SCT even if CR is not achieved. Sequential conditioning regimens have been shown to be a valuable option for patients with refractory disease, providing an alternative treatment approach for AML patients who do not respond well to initial therapies [50, 51]. Prospective Trials have been designed and are investigating the following at the moment (Table 2).

**Table 2:** List of all clinical trials assessing venetoclax in association with other agents in AML and MDS

Agents	Population	Phase study	References	Status
Venetoclax + CPX-351 (V-FAST)	ND AML fit for IC	Ib	NCT04075747	Active, not recruiting
Venetoclax + Azacitidine (Verona)	HR-MDS	III	NCT04401748	Active, not recruiting
Venetoclax + Evorpacept + Azacitidine	ND AML fit for IC	I/II	NCT04755244	Active, not recruiting
Venetoclax + CYC065 CDK Inhibitor	R/R AML	I	NCT04017546	Active, not recruiting
Venetoclax + SAB	High risk MDS	II	NCT04812548	Active, not recruiting
Venetoclax + MBG453 + Azacitidine	ND AML unfit for IC	II	NCT04150029	Active, not recruiting
Venetoclax + Pevonedistat + Azacitidine	ND AML unfit for IC	II	NCT04266795	Active, not recruiting
Venetoclax + Azacitidine + Pevonedistat	R/R AML	I	NCT04172844	Active, not recruiting
Venetoclax + ASTX727	AML	I/II	NCT04657081	Active, not recruiting
Venetoclax + Quizartinib	R/R FLT3 mutated AML	I/II	NCT03735875	Active, not recruiting
Venetoclax + STAT Inhibitor OPB-111077 + Decitabine	ND AML or R/R AML	I	NCT03063944	Active, not recruiting
Venetoclax + Nivolumab + Decitabine	ND AML	I	NCT04277442	Active, not recruiting
Venetoclax + Ruxolitinib	R/R AML	I	NCT03874052	Active, not recruiting
Venotoclax + (7 + 3)	ND AML and MDS-EB	III	NCT04628026	Active, recruiting
Venetoclax + Azacitidine or Decitabine	ND AML fit for IC	Ib	NCT03941964	Completed (No results posted)
Venetoclax + CC-90011 + Azacitidine	ND AML fit for IC	I	NCT04748848	Completed (No results posted)
Venetoclax + CPX-351	ND AML unfit for IC	Ib	NCT04038437	Completed (No results posted)
Venetoclax + Alvocidib	R/R AML	I	NCT03441555	Completed (No results posted)
Venetoclax + LDAC + Milademetan tosylate	R/R AML	I/II	NCT03634228	Completed (No results posted)
Venetoclax + azacitidine ± donor lymphocyte infusion	MDS or AML in Relapse after AHSCT	I/II	NCT05226455	Not yet recruiting
Venetoclax + LINT-AC225 + Azacitidine	R/R AML	I/II	NCT03932318	Not yet recruiting
Venetoclax + Decitabine	Young ND AML	III	NCT05177731	Recruiting
Venetoclax + ATRA + Azacitidine	ND AML fit for IC	III	NCT05654194	Recruiting
Venetoclax + Cytarabine + Mitoxantrone (RELAX)	R/R AML	I/II	NCT04330820	Recruiting
Venetoclax + BSR-236	ND AML unfit for IC	I/II	NCT05503355	Recruiting
Venetoclax + GO	R/R AML	Ib	NCT04070768	Recruiting
Venetoclax + TAGR + AZA	ND & R/R AML, MDS or BPDCN	Ib	NCT03113643	Recruiting
Venetoclax + LINT-AC225	R/R AML	I/II	NCT03867682	Recruiting
Venetoclax + CA-4948	R/R AML and high risk MDS	I/II	NCT04278768	Recruiting
Venetoclax + Voruciclib	R/R AML	I	NCT03547115	Recruiting
Venetoclax + Chidamide + Azacitidine	R/R AML	II	NCT05305859	Recruiting
Venetoclax + Siremadlin + Azacitidine	ND AML unfit for IC	I/II	NCT05155709	Recruiting
Venetoclax + Tamibarotene + Azacitidine	ND AML	II	NCT04905407	Recruiting
Venetoclax + CACAG Regimen	ND AML	I	NCT05659992	Recruiting
Venetoclax + Azacitidine + Homoharringtonine	R/R AML	III	NCT05457361	Recruiting
Venetoclax + Gemtuzumab ozogamicin (anti-CD33)	R/R AML	I	NCT04070768	Recruiting
Venetoclax + pegcrisantaspase	R/R AML	I	NCT04666649	Recruiting
Venetoclax + HMA + Aclarubicin	ND AML unfit for IC	III	NCT05264883	Recruiting
Venetoclax + ADI-PEG 20 + Azacitidine	AML	I	NCT05001828	Recruiting
Venetoclax + Cladribine + LDAC + Azacitidine	ND AML	II	NCT03586609	Recruiting
Venetoclax + Bomedemstat	R/R AML	I	NCT05597306	Recruiting
Venetoclax + IMGN632 (anti-CD123)	R/R AML	Ib/II	NCT04086264	Recruiting
Venetoclax + Uproleselan + Azacitidine	ND AML	I	NCT04964505	Recruiting
Venetoclax + Navitoclax + Decitabine	R/R AML to Venetoclax	I	NCT05222984	Recruiting
Venetoclax + 8-Chloroadenosine	R/R AML	I	NCT05263284	Recruiting
Venetoclax + Decitabine + Cedazuridine	R/R AML	II	NCT04975919	Recruiting
Venetoclax + CLAG-M	AML	I	NCT04797767	Recruiting
Venetoclax + Pitavastatin	AML	I	NCT04512105	Recruiting
Venetoclax + Selinexor	R/R AML	I	NCT04898894	Recruiting
Venetoclax + Azacitidine + Trametinib	AML or HR-MDS	II	NCT04487106	Recruiting
Venetoclax + Salsalate + Decitabine or Azacitidine	AML or MDS	II	NCT04146038	Recruiting
Venetoclax + Omacetaxine	R/R AML or MDS	I/II	NCT04874194	Recruiting
Venotoclax + (7 + 3)	ND AML	Ib	NCT03709758	Suspended
Venetoclax + S64315 (MCL1i)	R/R AML	I	NCT03672695	Terminated
Venetoclax + Alvocidib	R/R AML	II	NCT03969420	Terminated
Venetoclax + AZD5991	R/R AML	I	NCT03218683	Terminated
Venetoclax + AMG 176	R/R AML	I	NCT03797261	Terminated
Venetoclax + Azacitidine or Decitabine	ND AML fit for IC	Ib	NCT04454580	Unknown

In a recent subgroup analysis study by Dinardo et al. [52], it was discovered that among AML patients receiving venetoclax-based therapies, the strongest molecular associations with response were found to be present in patients with mutations in genes such as IDH1, IDH2, NPM1, and DNMT3A. These patients exhibited CR/CRi rates that exceeded 80%. The study also highlighted that the majority of patients with either NPM1 or IDH2 mutations were found in the durable remission cohort. Furthermore, the median OS for patients with either NPM1 or IDH2 mutations was not reached, with 2-years OS rates of 71.8% and 79.5%, respectively. The association between IDH1 mutations and prognosis, however, was found to be less clear, with no significant difference in OS observed. The research also established that resistance to venetoclax treatments is primarily caused by activated kinases and TP53 perturbation, with different signaling pathways activated by kinase activations, including RAS, FLT3-ITD, FLT3-TKD and CBL mutations [52].

Venetoclax-based combination therapies have demonstrated efficacy and tolerability, and the identification of mutations for which various drugs have been approved has spurred active investigation of venetoclax in combination trials. The use of multiple drug combinations may provide enhanced and sustained responses through synergistic effects and the targeting of multiple subclones of AML. Additionally, the combination of azacitidine and venetoclax can now bring hope to transplant patients who are unfit for IC. Although the quality of response achieved with this regimen may not always be as good as that of IC, the chances for a cure with allo-SCT in patients with CRi or PR are significant. Whereas these patients may experience a higher risk of non-relapse mortality, the risk of relapse is not significantly increased [18]. Herein, further phase I and II clinical trials are ongoing to thoroughly assess the use of venetoclax in AML patients (Table 2).

### Venetoclax Dose and Duration Adjustment

Patients receiving venetoclax-based therapies are subject to impaired hematopoietic functions, which are responsible for contracting fungal infections, in addition to non-hematopoietic adverse events (AEs) [53]. Azole antifungals are administered on a treatment or prophylaxis basis to these patients. The azoles family are strong cytochrome P450 3A inhibitors. Administration of venetoclax, in combination with certain azole-class like fluconazole, voriconazole, or posaconazole, may elicit clinically significant drug–drug interactions (DDIs) that have the potential to impact the therapeutic efficacy and safety of the treatment regimen. Pharmacokinetic studies have demonstrated that the concurrent use of venetoclax with azoles results in an elevation of venetoclax plasma concentrations, increasing the risk of toxicity [54].

It is crucial to closely observe patients undergoing treatment with venetoclax in conjunction with antifungal medications, and make adjustments to the dosage as necessary to minimize the risk of DDIs [55]. The recommended dosages for the administration of venetoclax in combination with HMA or chemotherapy is 400 mg/day, and 600 mg/day when used with LDAC [56]. As more comprehensive pharmacokinetic data becomes available, and until the capability to measure venetoclax concentrations in a clinical setting is achieved, it is recommended to adjust its dosage following azole usage as demonstrated in the VIALE-A and VIALE-C trials. This includes a reduction to 200 mg/day in conjunction with fluconazole, 50 or 70 mg/day with Posaconazole, and 100 mg/day with other potent CYP3A4 inhibitors such as voriconazole [57].

Posaconazole remains the only drug that has been studied regarding its dose adjustment. Using physiologic-based pharmacokinetics modeling analysis, it was found that a 70 mg daily dose of venetoclax is appropriate when administered with posaconazole’s recommended dose of 300 mg daily, up to 500 mg daily. This escalation led to only 12% increase in the median predicted exposures of venetoclax, which falls well within its safety margin limit [58]. Compared to administering a dose of 400 mg alone, co-administration of venetoclax at a 50-mg dose with multiple doses of posaconazole led to an increase in the mean venetoclax *C*_max_ and AUC_0–24_ by 53% and 76%, respectively. Furthermore, co-administration of venetoclax at 100 mg with posaconazole resulted in a 93% and 155% increase in the mean venetoclax *C*_max_ and AUC_0–24_, respectively. Upon adjusting for different doses and nonlinearity, posaconazole was found to increase venetoclax *C*_max_ and AUC_0-24_ by 7.1- and 8.8-fold, respectively [57].

Different durations of venetoclax exposure in venetoclax-based regimens have been investigated, given the AEs that are associated with its use, and their potential to cause dose interruption. For example, in a phase-I clinical trial, venetoclax was combined with the standard 7 + 3 induction regimen for patients with ND AML. The trial demonstrated that a daily dose of 200 mg for four days was safe for patients aged 18–60 [59]. Similarly, another study evaluated the combination of venetoclax with FLAG-IDA for adult patients with ND or R/R AML who are fit for chemotherapy. To maintain high response rates (RR) while mitigating AEs, the dosing regimen was adjusted by reducing venetoclax duration from 21 to 14 days and the cytarabine dose from 2 to 1.5 g. This modified regimen is currently recommended for phase-2 induction [60].

Some studies also explored 7, 14, and 21-day venetoclax administration with azacitidine. Willekens et al. [61] demonstrated that the azacitidine plus venetoclax combination with a shorter duration of venetoclax, only given concurrently with azacitidine for 7 days (7 + 7), can yield acceptable RR with less toxicity than the usual 7 + 28 dosing, particularly in good responders. This study was a multicenter (7 centers) retrospective study done on 82 untreated AML patients who were ineligible for intensive regimens and received both azacitidine and venetoclax for 7 days from the first cycle every 28 days. Median EFS and OS were 7.5 and 12.8 months, respectively, after a median follow-up of 4.8 months. Yet, even with the shorter venetoclax exposure, 88% of patients needed at least one transfusion, 49% experienced severe, grade III/IV, febrile neutropenia, and 48% needed a median delay of 13 days to start the second cycle. This study shows that although a shorter duration of venetoclax exposure in ND AML patients might not lead to complete toxicity control, it will lead to a more acceptable toxicity profile while maintaining efficacy [61].

Another study [62] on 13 untreated AML patients showed that a 14-day venetoclax administration with azacitidine compared favorably with the regular 28-day venetoclax administration. Venetoclax was given for 14 days to 61.5% of the patients (VEN14 group) and for the regular 28 days to the remainder (VEN28 group). Interestingly, the composite CR rate was not significantly different between the two groups (75% in the VEN14 group versus 80% in the VEN28 group). Even more so, while the median OS was reached in the VEN28 group after 254 days and the median EFS after 178 days, neither was reached in the VEN14 group. Another important finding regarding efficacy in this study was that patients in the VEN14 group achieved a higher rate of total WT1, an MRD marker, negativity than patients in the VEN28 group (50% versus 20%). This favorable MRD negativity outcome in the VEN14 group might have stemmed from the fact that although none of the patients in the VEN28 group was able to start cycle 2 as scheduled, secondary to myelosuppression, 50% of VEN14 patients did. Regarding toxicity, the VEN14 group experienced less febrile neutropenia (37.5% versus 80%) [62].

Finally, a study was done by Mirgh et al. [63] in 2021 in which the outcomes of 24 AML patients treated with azacitidine plus venetoclax combination as IC were explored. Because of hypocellular marrow occurring in two patients after induction with azacitidine plus venetoclax administered for 28 days, the induction protocol was amended to administering venetoclax for 21 days instead of 28. Even after that amendment, 66% of patients needed dose interruption given severe neutropenia. Hence, the protocol was further modified to give venetoclax at 400 mg once daily for 14 days only. The analysis showed that patients who received venetoclax for a shorter duration, less than 21 as opposed to 21–28 days, needed a lesser duration of antibiotics and achieved earlier count recovery while maintaining the same RR and myelosuppression severity. The authors went further and proposed that, given the same efficacy and faster count recovery, shorter venetoclax exposure can lead to more CR as opposed to CRi, which is known to be associated with worse prognosis and more complications [63].

Based on these results, shorter durations of venetoclax in venetoclax-based regimens can be the key to limiting the toxicity associated with these regimens. Nonetheless, further research is needed to determine the optimal duration of Venetoclax administration in these patients, given the retrospective nature and small sample size in the aforementioned studies.

### Toxicities and Adverse Events

The most common hematologic AEs associated with venetoclax-based therapies are neutropenia, thrombocytopenia, anemia, and fatigue, while the non-hematologic ones consist mainly of hypokalemia and GI manifestations. Additionally, some studies also reported an increased incidence of infections, bleeding, and tumor lysis syndrome. The prolonged utilization of venetoclax-based regimens is often associated with significant myelosuppression, and a majority of patients will likely require dose/duration adjustments of both venetoclax and the backbone therapy [64]. Management of hematological AEs in AML consists of delaying subsequent doses of venetoclax with LDAC or HMA, and the supplementation of G-CSF with antimicrobial prophylaxis in cases of the first occurrence of grade 4 neutropenia, with or without fever for more than 7 days, post-remission. In the second or subsequent occurrences, a similar protocol applies, with a reduction of the venetoclax treatment cycle by 7 days. No interruption in treatment protocol occurs if neutropenia happens prior to remission. A bone marrow biopsy should be considered in the case of persistent prolonged cytopenia or new onset cytopenia in the remission period [64]. As for non-hematological events, venetoclax should be interrupted at any occurrence, if grade 3–4 AEs do not resolve with supportive care. Specifically, for venetoclax + rituximab or obinutuzumab, there is no recommendation for dose reduction or delays of venetoclax in grade 1 events, while a delay of 28 days should be considered in grade 3–4 AEs in their first occurrence. As for subsequent episodes, one dose-level reduction of venetoclax can be implemented, for example reducing from 400 to 300 mg [64].

### Venetoclax Studies on MDS Patients

Results of venetoclax-based combination therapy in AML clinical trials (Table 1) elucidated a possible synergistic effect with HMA agents which sparked the start of similar trials on in MDS patients [27, 39]. One such study is the phase 1b trial (NCT02942290) on 78 treatment-naïve HR-MDS using venetoclax on an escalating dose: 100, 200, and 400 mg for 14 days out of a 28-day cycle plus azacytidine, 75 mg/m^2^, day 1 to 7. This study yielded an ORR of 77%, CR of 42%, marrow CR (mCR) of 35%, and a median OS of 27.5 months [39, 64]. One interesting finding was that 65% of transfusion-dependent patients became transfusion-independent. The most common side effects were neutropenia (51%) and thrombocytopenia (30%). Febrile neutropenia (42%) was the most common severe AE [39, 64]. As a consequence of these results, a phase III placebo-controlled trial (VERONA trial, NCT04401748) has started and is still recruiting HR-MDS patients. The future results of this trial will elucidate if the high mCR obtained in the phase Ib trial will translate into a survival advantage. Also, it will more accurately show whether the combination of venetoclax plus azacitidine is superior to placebo plus azacitidine.

The optimal dose and duration of venetoclax treatment in MDS is currently still an area of investigation, especially for elderly patients. While a recommended phase-2 dosage of 400 mg once daily for a 28-days cycle in combination with azacitidine has been established, it should be acknowledged that this is based on limited data, and further research is needed to fully determine the optimal dosing and duration of venetoclax in MDS patients [65].

Current clinical trials are evaluating the effectiveness of venetoclax combined with azacitidine for the treatment of R/R HR-MDS patients. Preliminary results from a phase 1b trial show that patients treated with venetoclax-based combination therapy, compared to a single-agent venetoclax, show a median response time of 1.2 months, an ORR of 40% and a 12-months OS estimate of 65%. A retrospective study on pre-treated HR-MS patients treated with venetoclax plus an HMA revealed an mCR rate of 59%, and 62% of the responsive patients underwent an allo-SCT [66]. This suggests that venetoclax-based combination therapy in MDS can serve as a bridge to the allo-SCT in the future [67].

### Resistance to Venetoclax

Venetoclax is establishing its transformative potential in the management of AML and MDS, improving the outlook for these diseases in high-risk and elderly patients. However, some of the latter are not responsive to venetoclax-based combination therapy [1]. Moreover, a proportion of the patients who achieve a response will experience relapse post-treatment. These relapsed or refractory disease patients have one thing in common: resistance. The prognosis in these patients is poor with CR/CRi of 13% and a median OS of 2.4 months [1].

Resistance to venetoclax can be primary or acquired throughout the treatment. Multiple mechanisms of resistance have been suggested in the literature. Clonal heterogeneity with multiple escape mechanisms is the most common [68, 69]. Some of these mechanisms are compensatory upregulation of myeloid cell leukemia-1 (MCL-1) and BCL-XL, an anti-apoptotic protein, after chronic venetoclax or prior HMA-exposure BCL-2 mutations, leading to reduced venetoclax binding and genomic instability [33, 70]. Moreover, patients with TP53-mutant AML and adverse cytogenetics or prior exposure to HMA have demonstrated lower RRs when treated with a combination of venetoclax and HMAs, 47% and 60% CR/CRi rates, respectively, resulting in a shorter duration of responses and poor survival of approximately 6 months. Thus, new approaches, possibly triplet combination regimens or novel targeted therapies, are required to address this issue [64].

Estimating the risk of resistance and developing preventative methods is critical. Some high-risk markers for resistance include FLT3, RAS, and TP53 mutations [70–72]. Also, treatment-related AML, secondary AML, and monocytic morphology were all associated with resistance. These mutations consistently showed higher minimal residual disease positivity and inferior ORR. On the other hand, MRD negativity, NPM1, IDH-1, or IDH-2 mutations all predicted better ORR and durability of the remission [34, 73].

### Tackling Resistance

Strategies to tackle or even prevent resistance should be developed. One possible option is the use of combination therapies with different mechanisms of action on the intrinsic apoptotic pathway [1]. Other targets in this apoptotic pathway include MCL-1 and BCL-XL [74]. Another strategy relies on the magnification of apoptosis by targeting the extrinsic apoptotic pathway through culprits such as MDM2 [74]. Last, but not least, venetoclax sensitizes cancer cells to traditional chemo, immuno and cell therapy by lowering the apoptotic threshold in response to cell stress, even without the presence of increased BCL-2 expression [1]. Hence, venetoclax in combination with chimeric antigen receptor T-cell therapy warrants consideration, given the fact that venetoclax is nontoxic to T-lymphocytes [75].

## Conclusion

Venetoclax-based combination regimens have proven that the therapeutic nihilism in the management of high-risk AML and MDS patients should be abandoned, given the positive outcomes of these combinations in front-line and salvage therapy. Some of the obstacles noted in the use of venetoclax in these diseases are toxicity, especially myelosuppression, infections, and resistance. Consequently, close monitoring for myelosuppression and the use of preventative supportive measures such as antibacterial, antiviral, and growth-stimulating factors are essential to fully realize the benefits of venetoclax. Moreover, shorter durations or lower doses may also offer a promising approach to surmounting these obstacles, while maintaining the positive outcomes associated with the use of venetoclax in AML and MDS patients.

## Data Availability

The data used in this review article is based on publicly available sources, including published research articles, books, and reputable online databases. No new data was generated or collected for the purpose of this review.
